# Temperature and Strain Rate Dependence on the Tensile Mechanical Properties, Constitutive Equations, and Fracture Mechanisms of MarBN Steel

**DOI:** 10.3390/ma16083232

**Published:** 2023-04-19

**Authors:** Yunqing Jiang, Tongfei Zou, Meng Liu, Yifan Cai, Quanyi Wang, Yunru Wang, Yubing Pei, Hong Zhang, Yongjie Liu, Qingyuan Wang

**Affiliations:** 1 Failure Mechanics and Engineering Disaster Prevention Key Laboratory of Sichuan Province, College of Architecture and Environment, Sichuan University, Chengdu 610065, China; 2 Key Laboratory of Deep Underground Science and Engineering, Ministry of Education, Sichuan University, Chengdu 610065, China; 3 State Key Laboratory of Long-Life High Temperature Materials, Dongfang Turbine Co., Ltd., Deyang 618000, China; 4 School of Architecture and Civil Engineering, Chengdu University, Chengdu 610106, China

**Keywords:** MarBN steel, tensile behavior, strain rate, temperature, constitutive models

## Abstract

The effect of strain rate and temperature on the thermomechanical behavior and microstructure of MarBN steel is studied with the strain rates of 5 × 10^−3^ and 5 × 10^−5^ s^−1^ from room temperature (RT) to 630 °C. At high strain rates of 5 × 10^−3^ s^−1^, the Holloman and Ludwigson equations can better predict tensile plastic properties. In contrast, under low strain rates of 5 × 10^−5^ s^−1^, coupling of the Voce and Ludwigson equations appears to predict the flow relationship at RT, 430, and 630 °C. However, the deformation microstructures have the same evolution behavior under strain rates and temperatures. Geometrically necessary dislocations appear along the grain boundaries and increase the dislocation density, which results in the formation of the low-angle grain boundaries and a decrease in the number of twinning. The strengthening sources of MarBN steel include grain boundary strengthening, dislocation interactions, and multiplication. The fitted R^2^ values of these models (JC, KHL, PB, VA, ZA) to plastic flow stress at 5 × 10^−5^ s^−1^ are greater than 5 × 10^−3^ s^−1^ for MarBN steel. Due to the flexibility and minimum fitting parameters, the phenomenological models of JC (RT and 430 °C) and KHL (630 °C) give the best prediction accuracy under both strain rates.

## 1. Introduction

To reduce carbon dioxide emissions, the ultra-supercritical (USC) power plant with increased steam temperatures and pressures can be applied worldwide. MarBN is martensitic 9Cr steel strengthened with boron and nitrides [1], which has been alloy-designed and subjected to long-term creep and oxidation loading conditions for application to thick section boiler components in USC power plants above 600 °C [2]. In MarBN, the high strength and oxidation originate from stabilizing lath martensitic embedded in the prior austenite grain boundaries (PAGBs) [3,4,5,6,7].

Many studies have shown that the microstructure of MarBN steel has a significant effect on its mechanical properties. The content of pre-eutectic carbides and the size of austenite grains can significantly affect their yield strength, tensile strength, and plastic properties [8]. It was also shown that creep in MarBN steels is similarly influenced by microstructures such as dislocations, precipitates, and structural boundaries [9]. In addition, the deformation behavior of MarBN steel under various loading conditions has been of interest to researchers. Fournier et al. [7] studied the soft behavior under cyclic loadings at high temperatures and proposed a model to describe the deformation microstructure at the scale of slip systems. Zhang et al. [3] reported the effect of temperatures on fatigue properties and damage mechanisms. They pointed out that cyclic softening is associated with the laths, dynamic recrystallization at room temperature (RT), and grain rotation at high temperatures. Furthermore, they compared and discussed the fatigue response and damage mechanisms under stress- and strain-controlled fatigue modes [10]. Xiao et al. [11] studied the high-temperature tensile behavior and fracture mechanisms of 9% chromium-tempered martensitic steel and described the tensile deformation using the hyperbolic sine law. However, to our knowledge, there are few systematic studies of MarBN steel based on the thermomechanical properties and deformation mechanism using electron backscattered diffraction (EBSD) techniques and constitutive models regarding the considered effect of both strain rates and temperatures. Consequently, the effect of strain rates and temperatures on the microstructure of plastic deformation is still unknown.

Furthermore, constitutive models have been used to simulate and understand the plastic flow stress behaviors of alloys, including phenomenological and physically based models. The JC [12] and KHL [13] models are well-known phenomenological models that can be applied to various materials [14,15,16]. PB [17,18,19], VA [20], and ZA [21] form another group of physically based models that are related to the micromechanics of plastic deformation. To understand the plastic deformation behavior and simulate the flow stress, selection and modification within existing constitutive models are necessary and efficient. Therefore, in this study, it is necessary to compare the predictive capabilities and accuracy among these constitutive models under different thermomechanical loading conditions for MarBN steel.

In the current investigation, this paper focuses on three purposes. The first is to study the thermomechanical properties and deformation mechanism of MarBN steel under various temperatures and strain rates. The second is to compare and evaluate the deformation microstructure with wide temperatures and strain rates. The third is to compare and analyze the predictive capabilities for different constitutive models under thermomechanical tensile loading conditions for MarBN steel. Uniaxial tensile tests are conducted over a wide range in temperature (room temperature (RT), 430, 630 °C) and strain rate (5 × 10^−3^ and 5 × 10^−5^ s^−1^). Emphasis is applied to the effect of temperature and strain rate on the flow stress and work-hardening behavior. Deformation microstructures are observed and evaluated to understand the plastic evolution and failure mechanisms using electron backscattered diffraction (EBSD) techniques. Finally, several constitutive models are used to simulate the flow stress and understand the effect of temperatures and strain rates on the flow stress.

## 2. Materials and Methods

The MarBN steel [1] used in the current study was received in the heat treatment condition, such as 1250 °C/2 h + 1300 °C/2 h + air cooling (AC), and the aging treatment, i.e., Ⅰ: 1150 °C/6 h + AC; Ⅱ: 850 °C/20 h + AC. It has the nominal composition (wt. %) as follows, 0.10 C, 9.16 Cr, 0.06 Si, 0.20 Mn, 0.20 Mo, 0.40 Ni, 0.08 Nb, 2.95 W, 2.82 Co, 0.20 V, and Fe as balance [3]. A uniaxial tensile test was conducted with the constant strain rates of 5 × 10^−3^ and 5 × 10^−5^ s^−1^ under different temperatures of RT, 430 °C, and 630 °C, using a mechanical testing system with a 100 kN load cell equipped resistance furnace with the temperature fluctuation at ±5 °C based on the international standard of tensile testing [22,23]. Cylindrical specimens 5 mm in diameter and 10 mm in length were used to test the tensile properties, such as yield and ultimate tensile strength. The extensometer was used to measure the strain during tensile tests. For high-temperature testing, the specimen was heated up to a high temperature for 20 min to obtain a uniform and constant temperature. The test was repeated three times to minimize the error caused by the data scatter.

Before performing EBSD orientation characterization, the samples were mechanically polished with 400#, 1000#, 2000#, and 3000# SiC water-abrasive paper. Then, the samples were electropolished to remove the surface deformation layer produced with mechanical grinding. The polishing solution composition was 7% perchloric acid and 93% ethanol, and the current density was 450 mA/cm^2^ for about 1 min. EBSD scans were conducted using the FEI Quanta 450F field emission scanning electron microscope (SEM) coupled with an Oxford HKL system. The acceleration voltage of EBSD was 15 kV, and the scan step size range was 0.2 μm. Image quality (IQ), grain boundaries (GBs), grain misorientation, and inverse pole figure (IPF) were directly obtained using AZtecHKL software (Version: 5.12.63.0). To address the plastic strain evolution and dislocation density, the MTEX toolbox [24] in Matlab is used and solved to obtain the kernel average misorientation (KAM) [25] and geometrically necessary dislocations (GNDs) [26,27].

To address deformation mechanisms in the macroscopic stress–strain response, two phenomenological models (JC and KHL) and three physically based models (PB, ZA, and VA) were used to simulate and represent the plastic behavior considering the strain rate and temperature during the tensile process for MarBN steel. The experimental data fitting defines the initial values of the model’s coefficient, which is then optimized with experimental data.

## 3. Results and Discussion

### 3.1. Mechanical Response during Uniaxial Tensile

#### 3.1.1. Effect of Strain Rate and Temperature on the Flow Stress Behavior

**Figure 1:**
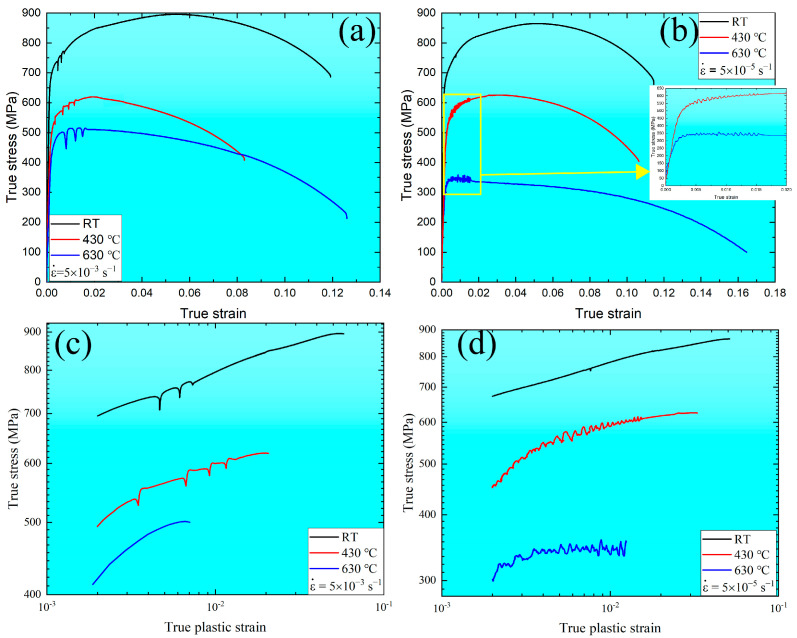
Effect of strain rates and temperatures on the true stress–true strain and true stress–true plastic strain for MarBN steel: (**a**,**c**) at 5 × 10^−3^ s^−1^ and (**b**,**d**) at 5 × 10^−5^ s^−1^.

The true stress–true strain curves as a function of strain rate and temperature are presented in Figure 1a,b. At a constant strain rate, the true stress curves divide into three stages: rise rapidly before the yield strength, the work-hardening period between yield and ultimate tensile strength, and the rapid descent failure stage above the ultimate tensile strength. The yield and ultimate tensile strength decrease with increasing temperature. More details are provided in Table 1. Serrated flows [28] are observed under all temperatures at the high strain rate of 5 × 10^−3^ s^−1^ and under 430 and 630 °C at the low strain rate of 5 × 10^−5^ s^−1^. At a high strain rate of 5 × 10^−3^ s^−1^, the serrated flows show a true stress–true strain curve followed by continuous drops below the general level of true stress–true strain values, defined as type C serrations [29,30]. However, at a low strain rate of 5 × 10^−5^ s^−1^, the serrated flows are defined as type A+B serrations [29,30,31]. In contrast, the type A serrations [32] are characterized as rising rapidly in the true stress–true strain curve, resulting in discontinuous drops to or below the general level of the true stress–true strain curve. Type B serrations [33] are oscillated along with the general level of the true stress–true strain curve. These observations indicate that the serrated flow behavior and type are related to the strain rate and temperature for MarBN steel. In addition, the true stress–true plastic strain (from yield strength to ultimate tensile strength) behavior for the high and low strain rates of 5 × 10^−3^ s^−1^ and 5 × 10^−5^ s^−1^ at RT, 430, and 630 °C are presented as double logarithmic plots, as shown in Figure 1c,d. The true plastic strain ranges for both temperatures decrease with increasing temperatures, resulting from the softening behavior at high temperatures for MarBN steel. The serrated flows can be observed along with the true plastic strain process, except for the strain rate of 5 × 10^−3^ s^−1^ at 630 °C. This result shows that the serrations occur above the ultimate tensile strength for a strain rate of 5 × 10^−3^ s^−1^ at 630 °C, resulting from the balance in the interaction between dislocation and solute atoms and softening behavior. At RT, the curves at both strain rates exhibit curvilinear behavior for all true plastic strains.

**Figure 2:**
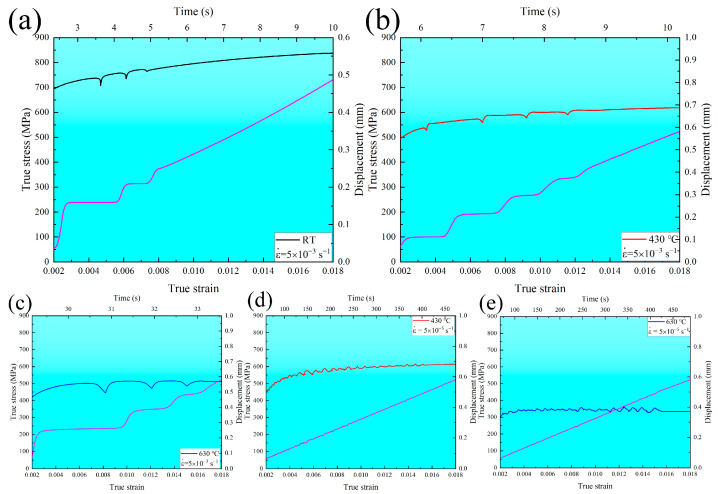
True stress–true strain curves and displacement vs. time (purple line) for MarBN steel of strain rate of 5 × 10^−3^ s^−1^ at (**a**) RT, (**b**) 430 °C, (**c**) 630 °C; and 5 × 10^−5^ s^−1^ at (**d**) 430 °C, (**e**) 630 °C.

Based on the previous reports [33,34,35,36], serrated flow results from movement in the dislocations along with the slip path caused by the interaction between dislocations and solute atoms. In the current study, the applied strain for the specimen was determined using the gauge displacement monitored with the extensometer during uniaxial tensile tests. The time and displacement curves concomitant with the serrations are presented in Figure 2. Each abrupt rise in the time vs. displacement curves indicates a transient and high localized deformation behavior. The abrupt rise in the curves under all temperatures at the high strain rate of 5 × 10^−3^ s^−1^ is more evident than that under 430 and 630 °C at the low strain rate of 5 × 10^−5^ s^−1^. Therefore, the type C serrations are mainly controlled by the strain rate, especially at a high strain rate, which causes more intense deformation per unit time, resulting in the local deformation bands possibly occurring outside the gauge length. However, the type A+B serrations are determined by the temperature, especially under high temperatures at a low strain rate. At a low strain rate of 5 × 10^−5^ s^−1^, the number and velocity of dislocations increase slowly. Thus they do not achieve a critical moving speed of serration initiation and cannot break away from the atmosphere of solute atoms [37,38]. When the temperature increases, the diffusion of solute atoms increases, improving the interaction between dislocation and solute atoms. Then, the dislocations can break away from the atmosphere of solute atoms, resulting in the multiplication of dislocation along with the slip path and, finally, the formation of type A+B serrations.

#### 3.1.2. Work-Hardening Parameters Dependent on the Strain Rate and Temperature

**Figure 3:**
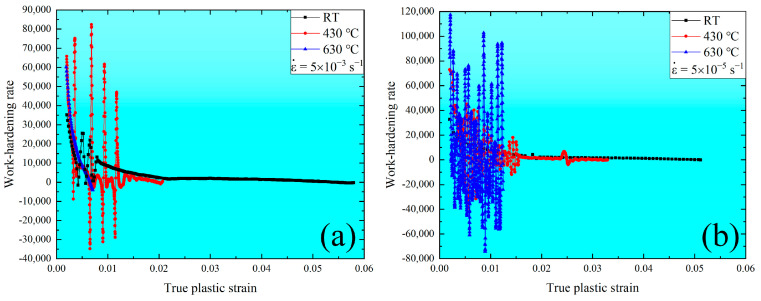
Work-hardening rate vs. true strain under different temperatures at the strain rate of 5 × 10^−3^ s^−1^ at (**a**) and 5 × 10^−5^ s^−1^ at (**b**).

The work-hardening region starts as the strain increases beyond the yield strength point and ends at the ultimate strength point during the uniaxial tensile stress–strain curve, which is associated with the dislocation generation, accumulation, and multiplication along with the grain boundaries. According to Figure 1, the work-hardening rate vs. true plastic strain under different temperatures at the strain rates of 5 × 10^−3^ s^−1^ and 5 × 10^−5^ s^−1^ are plotted, respectively, as shown in Figure 3. In contrast, the work-hardening rate decreases with increasing the true plastic strain up to about 0.06. The serration behavior under RT and 430 °C at a strain rate of 5 × 10^−3^ s^−1^ is present along with the work-hardening rate curves. The same is found under 430 and 630 °C at a strain rate of 5 × 10^−5^ s^−1^. These phenomena indicate that work hardenability is sensitive to strain rate and temperature.

In order to study the effect of strain rate and temperature on the true stress–true strain behavior, several flow relationships in the literature can be used, such as Hollomon (Equation (1)) [39], Ludwik (Equation (2)) [40], Swift (Equation (3)) [41], Ludwigson (Equation (4)) [42], and Voce (Equation (5)) [43,44], as follows,

(1) $$\sigma =K_{1}\varepsilon^{n_{1}}$$

(2) $$\sigma =\sigma_{y}+K_{2}\varepsilon^{n_{2}}$$

(3) $$\sigma =K_{3}{\left(\varepsilon_{0}+\varepsilon \right)}^{n_{3}}$$

(4) $$\sigma =K_{4}\varepsilon^{n_{4}}+\exp\left(K_{5}+n_{5}\varepsilon \right)$$

(5) $$\sigma =\sigma_{s}-\left(\sigma_{s}-\sigma_{1}\right)\exp\left[\frac{-\left(\varepsilon -\varepsilon_{1}\right)}{\varepsilon_{c}}\right]$$

where σ is the true stress; ε is the true plastic strain; $K_{1}$ and $n_{1}$ are the strain hardening coefficient and exponent in the Hollomon power-law relationship, respectively; $\sigma_{y}$ is the yield stress; $K_{2}$ and $n_{2}$ are the strain hardening coefficient and exponent in Ludwik relationship, respectively; $\varepsilon_{0}$ is the addition of pre-strain; $K_{3}$ and $n_{3}$ are the strain hardening coefficient and exponent in the Swift relationship, respectively; $K_{4}$ and $n_{4}$ are the same as in the Holloman relationship (Equation (1)), respectively; $K_{5}$ and $n_{5}$ are additional constants in the Ludwigson relationship, respectively; $\sigma_{s}$ is the saturation stress; $\varepsilon_{c}$ is a constant; and $\sigma_{1}$ and $\varepsilon_{1}$ are the true stress and true plastic strain in the Voce relationship, respectively.

In the present work, the least-squares method [45] was used to fit the parameters in Equations (1)–(5), as shown in Table 2. Furthermore, to prove the ability of different flow relationships for MarBN steel during uniaxial tensile, typically $R^{2}$, the sum of the square of the absolute difference between calculated stress values and the experimental values [46] for various flow relationships (Equations (1)–(5)) under different temperatures at the strain rates of 5 × 10^−3^ s^−1^ and 5 × 10^−5^ s^−1^ are computed in Table 2. Generally, the $R^{2}$ values fitted with Equations (1)–(5) at the high strain rate of 5 × 10^−3^ s^−1^ are higher than at the low strain rate of 5 × 10^−5^ s^−1^. For a strain rate of 5 × 10^−3^ s^−1^, all the $R^{2}$ values fitted with Equations (1)–(5) decrease with increasing temperatures, resulting from the decrease in the positive stress deviations under high temperatures [47]. However, the Holloman and Ludwigson equations calculate higher $R^{2}$ values under all the temperatures. The difference between Holloman and Ludwigson is very small. At the strain rate of 5 × 10^−5^ s^−1^, the Voce equation displays the highest $R^{2}$ values under RT and 430 °C. As the temperature arrives at 630 °C, the Ludwigson equation calculates the highest $R^{2}$ values. Similar results were reported for other types of steel [36,47,48,49]. Therefore, combining Voce (RT and 430 °C) and Ludwigson (630 °C) can provide the best description of true stress–true plastic strain behavior. To sum up, these results indicate that Equations (1)–(5) can better fit the flow relationship at the high strain rate of 5 × 10^−3^ s^−1^; especially, the Holloman and Ludwigson equations provide a useful prediction of tensile plastic properties for MarBN steel. When the strain rate decreased to 5 × 10^−5^ s^−1^, coupling the Voce and Ludwigson equations appears to predict the flow relationship at RT, 430, and 630 °C.

### 3.2. Microstructural Evaluation under Different Degrees of Deformation

#### 3.2.1. EBSD Maps and Analyses at a Strain Rate of 5 × 10^−3^ s^−1^ under RT, 430 °C, and 630 °C

**Figure 4:**
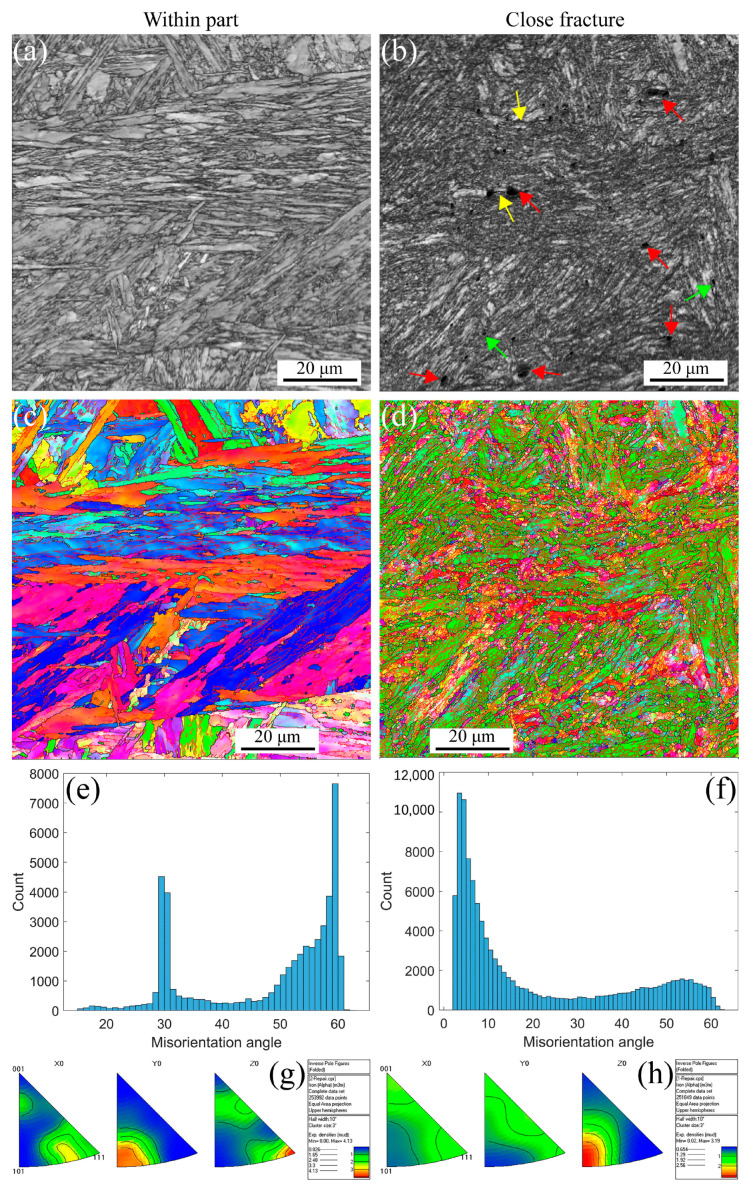
EBSD maps under different strain gradients at the strain rate of 5 × 10^−3^ s^−1^ under RT (Red arrows indicate voids within the grain and along the line, green arrows indicate shrinking microstructures, and yellow arrows point to voids and shrinking cracks). (**a**,**b**) IQs; (**c**,**d**) IPFs; (**e**,**f**) misorientation angles; and (**g**,**h**) inverse polo figures.

Figure 4 shows the EBSD maps, including IQs, IPFs, misorientation angles, and inverse polo figures at the strain rate of 5 × 10^−3^ s^−1^ under RT. The deformation microstructure presents at different positions, such as within part, and closes fracture due to the strain gradient during the tensile process. The stable PAGBs and martensite laths observed in Figure 4a,c are the same as the initial microstructure. When the strain gradient increases, the PAGBs and laths become unstable, forming the small, distorted, and elongated microstructure, as shown in Figure 4b,d, resulting in the formation of defects, i.e., voids within and along the grains indicated with the red arrows, shrinkage microstructure defined with the green arrows, and cracks across the voids and shrinkage marked with the yellow arrows. The distribution of misorientation angles at different strain gradients is shown in Figure 4e,f. The angle is a high-angle distribution of 30 and 50–60 degrees within part of the low-strain gradient. In contrast, with a close fracture for a high-strain gradient, the angle transforms into a low-angle distribution of 10 degrees. These results are in agreement with the results in Figure 4a–d because the high-strain gradient leads to distorted and elongated grains. Essentially, dislocations increase movement along the slip paths under plastic strain and multiplicate along the barrier [50,51], forming the LAGBs. Furthermore, in Figure 4g,h, the apparent texture characterization can be observed along the <101> direction. As the strain increases, the texture becomes more pronounced. At low strain, there is a texture distribution in both <101>//Z_0_ and <111>//X_0_, and as the strain increases, there is only a clear texture in the <101>//Z_0_ direction, and the strength of the texture decreased from 4.13 to 3.19. These results indicate that the material orientation becomes uniform after high plastic strain during the uniaxial tensile process at RT for MarBN.

**Figure 5:**
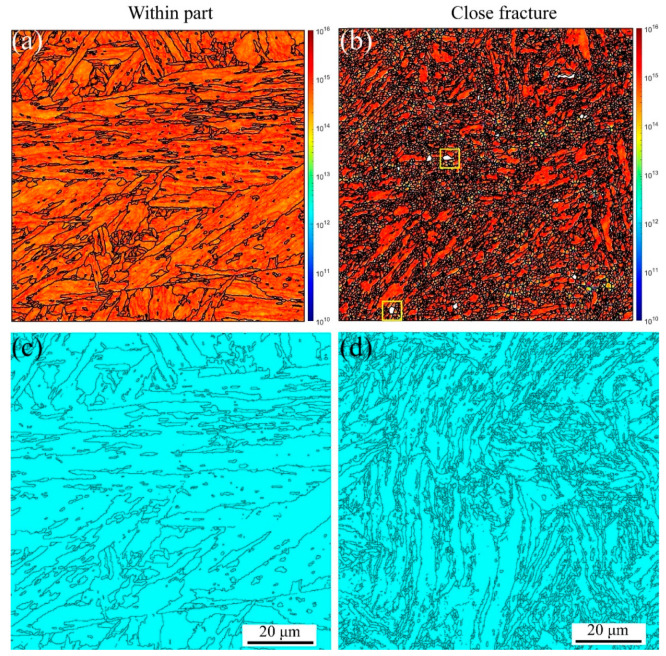
KAM, GND, and twinning maps under different strain gradients at the strain rate of 5 × 10^−3^ s^−1^ under RT (Voids are inside the yellow square). (**a**,**b**) GNDs and (**c**,**d**) twinnings.

On the other hand, the GND and twinning maps are presented in Figure 5. From Figure 5a,b, significant GND accumulations are present along the grain boundaries and triple junctions, resulting from the abrupt slip paths at the grain boundaries [52,53]. However, the GND densities are lower within the grain interiors. With increasing plastic deformation, the GND density increases. These results agree with Ashby’s GND model [27,53], indicating that the strain gradients and GND density are proportional to the applied strain. The yellow rectangles in Figure 5b are the voids, which are the same as those shown in Figure 4b, marked with red arrows. In Figure 5c,d, the twinning evolution can be observed, such as 4.8% within the part and 0.23% close fracture, indicating the HAGBs in the initial material cause many annealing twinnings. With increasing the plastic strain, the annealing twinnings disappeared due to the increase in the LAGBs caused by the dislocation densities, as shown in Figure 4f. Therefore, tensile failure mechanics is mainly controlled by the dislocation movement along the slip path for MarBN steel at RT.

**Figure 6:**
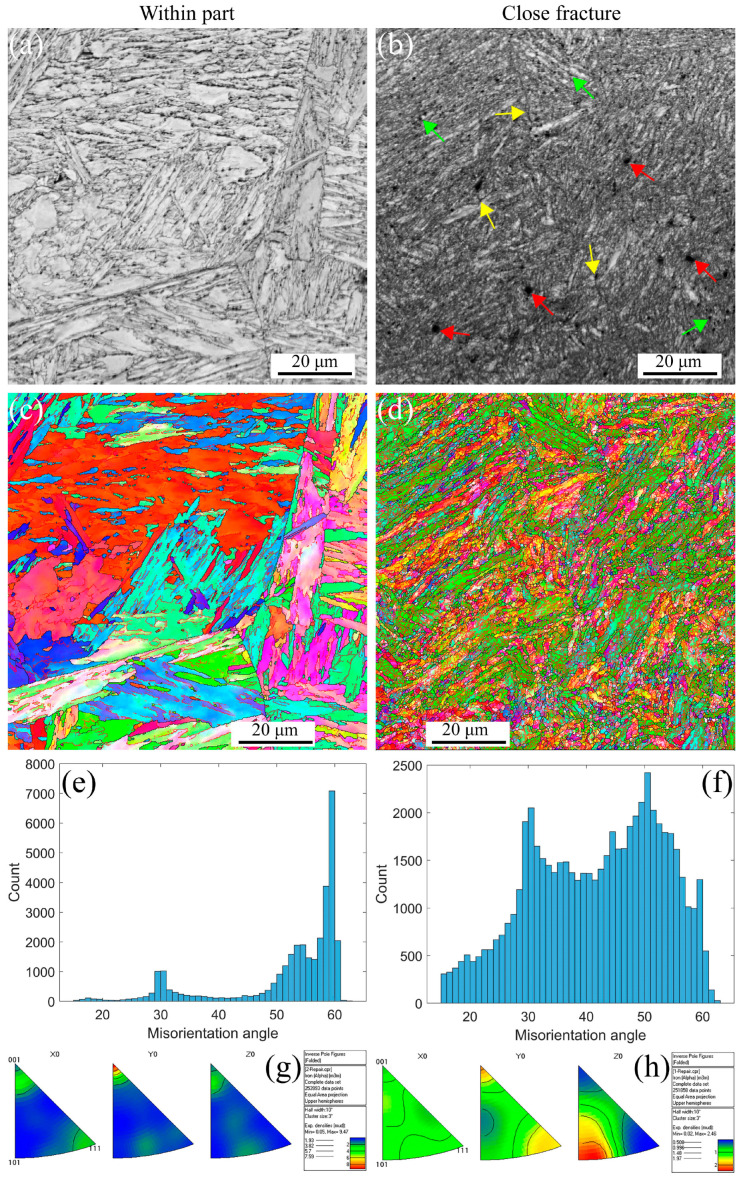
EBSD maps under different strain gradients at the strain rate of 5 × 10^−3^ s^−1^ under 430 °C (Red arrows indicate voids within the grain and along the line, green arrows indicate shrinking microstructures, and yellow arrows point to voids and shrinking cracks). (**a**,**b**) IQs; (**c**,**d**) IPFs; (**e**,**f**) misorientation angles; and (**g**,**h**) inverse polo figures.

Figure 6 shows the EBSD maps for the strain rate of 5 × 10^−3^ s^−1^ at 430 °C. The deformation microstructure presented at different positions is the same as the RT presented in Figure 4. However, the stable microstructure embedded in small voids along the grain boundaries is presented within the part in Figure 6a,c. In contrast, the deformation microstructure becomes unstable, and finally, the formation of the small, distorted, and elongated microstructure occurs, as shown in Figure 6b,d, resulting in the formation of the defects, i.e., voids indicated with the red arrows, shrinkage microstructure defined with the green arrows, and cracks through the voids and shrinkage marked with the yellow arrows. Figure 6e,f presents the distribution of misorientation angles at different strain gradients, in which the angle is a high-angle distribution of 50–60 degrees within part for a low strain gradient. In contrast, with a close fracture for a high strain gradient, the angle transforms into a low-angle distribution of 30–60 degrees. These results are different from the results in Figure 4e,f, where the misorientation angles are sensitive to the temperature. Certainly, the dislocation density decreases with increasing temperature [3,5,50,52], thus decreasing the number of LAGBs. However, the material orientation becomes uniform, as shown in Figure 6g,h, after high plastic strain during the uniaxial tensile process at 430 °C for MarBN.Same result as in RT, where there is significant texture in the high-strain region only in the <101>//Z_0_ direction. With increasing strain, the texture strength decreases from 9.47 to 2.46. This indicates that the plastic strain mainly controls the deformation orientation.

**Figure 7:**
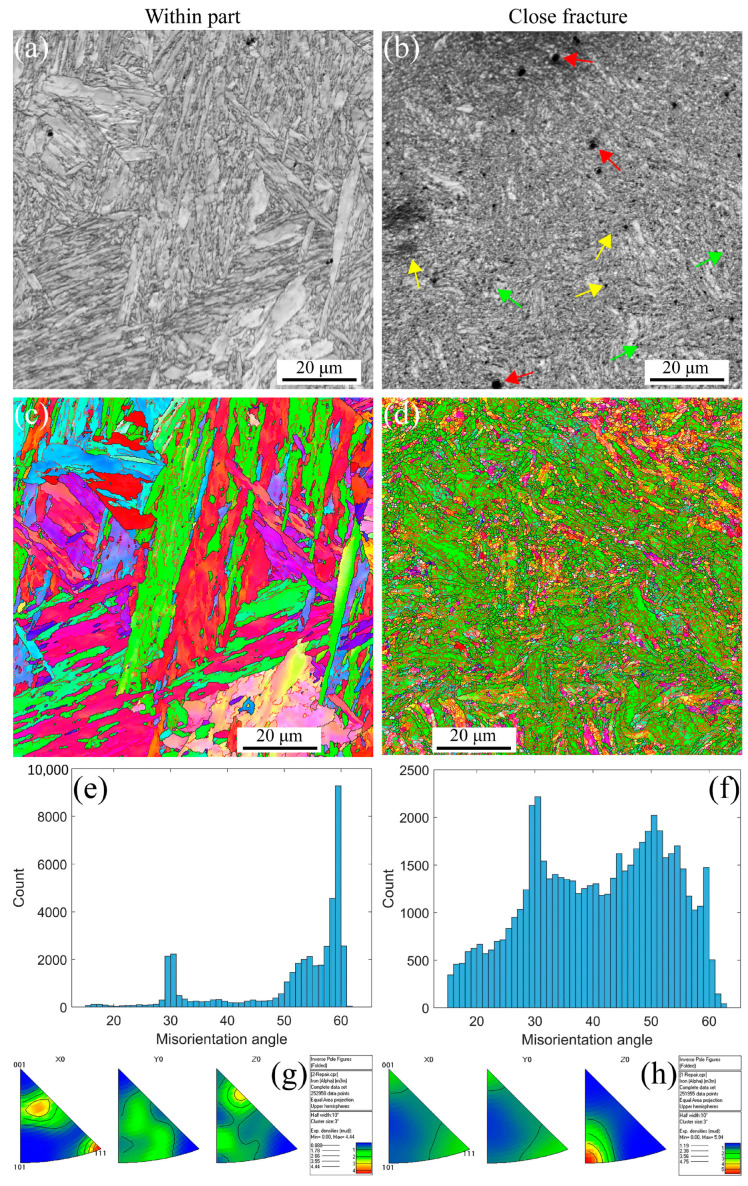
EBSD maps under different strain gradients at the strain rate of 5 × 10^−3^ s^−1^ under 630 °C (Red arrows indicate voids within the grain and along the line, green arrows indicate shrinking microstructures, and yellow arrows point to voids and shrinking cracks). (**a**,**b**) IQs; (**c**,**d**) IPFs; (**e**,**f**) misorientation angles; and (**g**,**h**) inverse polo figures.

Figure 7 shows the EBSD maps for the strain rate of 5 × 10^−3^ s^−1^ at 630 °C. The deformation microstructure presented at different positions is the same as that for RT and 430 °C, as presented in Figure 5 and Figure 6. However, the stable microstructure embedded in larger voids along the grain boundaries is observed within the part in Figure 7a,c due to the softening behavior at high temperatures. In contrast, the deformation microstructure becomes unstable, and finally, the formation of the small, distorted, and elongated microstructure occurs, as shown in Figure 7b,d, resulting in the formation of defects, i.e., voids indicated with the red arrows, shrinkage microstructure defined with the green arrows, and cracks through the voids and shrinkage marked with the yellow arrows. Figure 7e,f presents the distribution of misorientation angles at different strain gradients, which are the same as the results for 430 °C. These results also prove a critical temperature (CT) for the misorientation angles. For MarBN steel, before the CT, the misorientation angles are sensitive to temperature, as shown in Figure 6e,f. When exceeding the CT, the misorientation angles are insensitive to temperature and determined by the plastic strain. These results come from the balance between the dislocation decrease caused by the temperature and dislocation multiplication caused by the tensile plastic strain. However, the material orientation becomes uniform, as shown in Figure 7g,h, after high plastic strain during the uniaxial tensile process at 630 °C for MarBN, which is the same as the results for RT and 430 °C. Different from the other two temperatures, the texture strength did not decrease after larger strains but increased from 4.44 to 5.94. This is due to the softening in the material under tensile stress, where more grains aggregate to form stronger textures [54].

**Figure 8:**
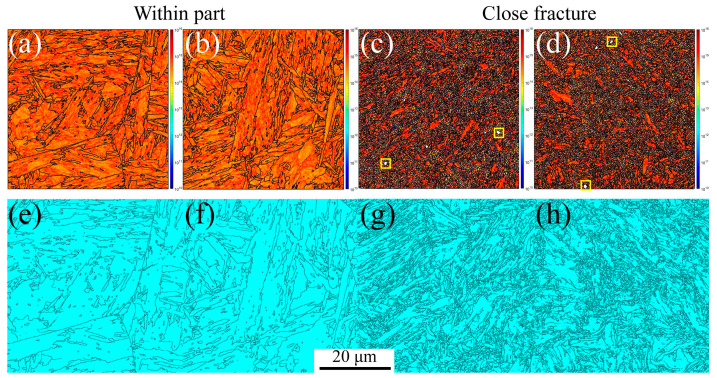
KAM, GND, and twinning maps under different strain gradients at the strain rate of 5 × 10^−3^ s^−1^ under 430 and 630 °C (Voids are inside the yellow square). (**a**–**d**) GNDs and (**e**–**h**) twinnings.

Furthermore, the GND and twinning maps at 430 and 630 °C are shown in Figure 8. The GND densities, as shown in Figure 8a–d, are the temperature and strain gradient balance. Furthermore, the results are the same as the results at RT, indicating that the effect of the strain gradient on the GND densities is more critical than the temperature during tensile loading conditions. In Figure 8e–h, the twinning evolution is presented as 6.45% and 5.98% at 430 and 630 °C, respectively, within the part, and 0.279% and 0.209% at 430 and 630 °C, respectively, close fracture, indicating that with increasing strain, the twinnings decrease. The higher the temperature, the lower the number of twinning. This means the number of twinning is related to the temperature and strain.

To sum up, at a strain rate of 5 × 10^−3^ s^−1^ under all temperatures, from the macro-perspective, with an increasing strain, the microstructure becomes unstable and then constitutes the deformed and elongated grain boundaries, resulting in the formation of the three defects defined as void, shrinkage, and crack. From the micro-perspective, GND appears along the GBs and increases the dislocation density. These result in the formation of the LAGBs and decrease the number of twinning. After significant strain, only <101>//Z_0_ texture exists at all three temperatures. This is because during the deformation process, the grains deform and rotate to balance larger stresses, resulting in irreversible deformation [55]. However, the texture strength at RT and 430 °C decreases compared to low strain, while it increases at 630 °C. This is usually related to the softening behavior of materials at high temperatures [3]. Therefore, the failure mechanics for MarBN steel are the result of the interaction between the macro- and micro-behavior.

#### 3.2.2. EBSD Maps and Analyses at a Strain Rate of 5 × 10^−5^ s^−1^ under RT, 430 °C, and 630 °C

**Figure 9:**
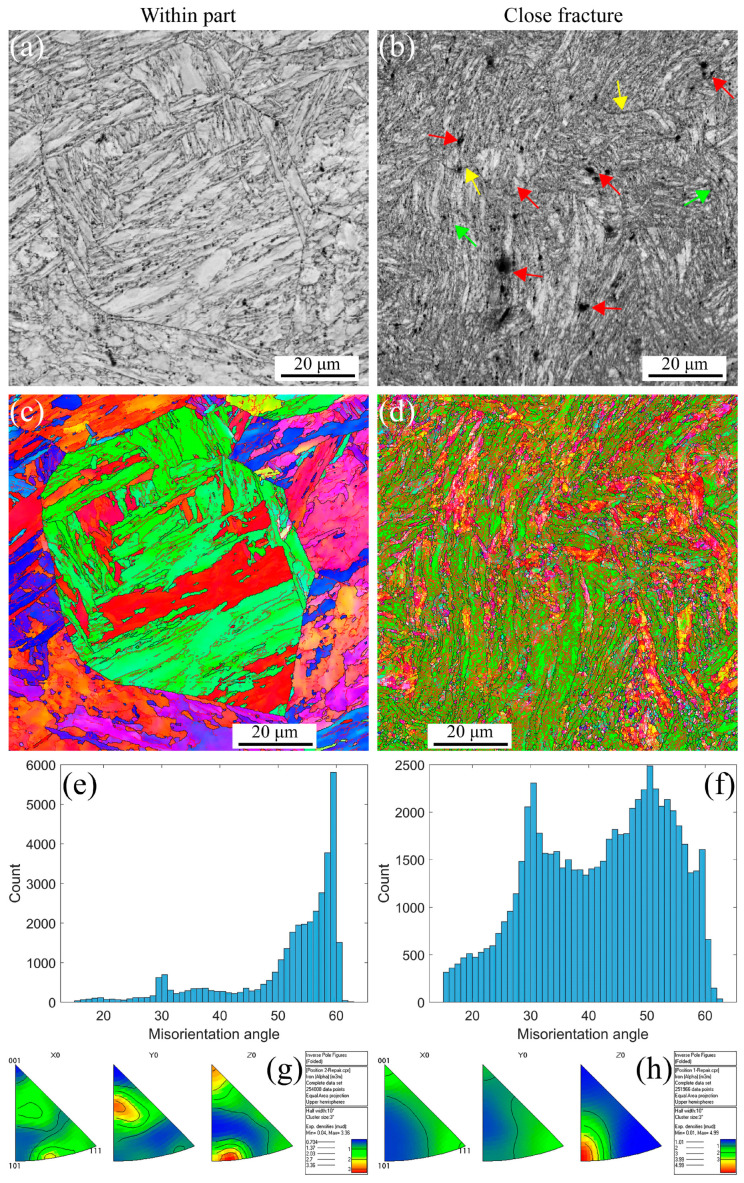
EBSD maps under different strain gradients at the strain rate of 5 × 10^−5^ s^−1^ under RT (Red arrows indicate voids within the grain and along the line, green arrows indicate shrinking microstructures, and yellow arrows point to voids and shrinking cracks). (**a**,**b**) IQs; (**c**,**d**) IPFs; (**e**,**f**) misorientation angles; and (**g**,**h**) inverse polo figures.

**Figure 10:**
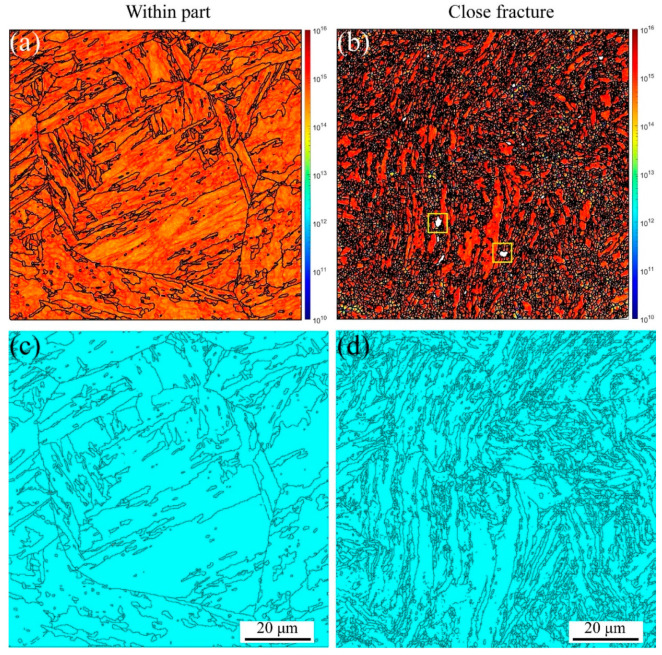
GND and twinning maps under different strain gradients at the strain rate of 5 × 10^−5^ s^−1^ under RT (Voids are inside the yellow square). (**a**,**b**) GNDs and (**c**,**d**) twinnings.

Similar results can be observed at a strain rate of 5 × 10^−5^ s^−1^ under RT, 430 °C, and 630 °C, as shown in Figure 9. The stable PAGBs and martensite laths are observed in Figure 9a,c. In contrast, the PAGBs and laths become unstable close fractures due to the severe plastic strain, and finally, the formation of the small, distorted, and elongated microstructure occurs, as shown in Figure 9b,d, leading to the formation of internal defects, i.e., voids indicated with the red arrows, shrinkage microstructure defined with the green arrows, and cracks across the voids and shrinkage marked with the yellow arrows. The misorientation angle is a high-angle distribution of 50–60 degrees within part for a low strain gradient. In contrast, with close fracture for a high strain gradient, the angle transforms into a 30–60 degree distribution, which is different from the angle distribution at the strain rate of 5 × 10^−3^ s^−1^ under RT. This indicates that the effect of the strain rate on the misorientation angle is crucial during tensile loading conditions for MarBN steel. Essentially, according to Orowan’s relationship [56], the strain rate and dislocation density are proportional as ${\dot{\varepsilon }}_{p}\propto \rho_{m}$ [57], in which the strain rate decreases and the dislocation density decreases, resulting in complex information about the LAGBs. Additionally, from Figure 9g,h, the material orientation becomes uniform after high plastic strain during the uniaxial tensile process under RT for MarBN, which is the same as the results at the strain rate of 5 × 10^−3^ s^−1^ under RT. Similarly, only the <101>//Z_0_ direction has significant texture, and the rest of the directions have no significant texture. However, when the strain rate is 5 × 10^−5^ s^−1^, the texture strength increases with the increase in plastic strain. On the other hand, the KAM, GND, and twinning maps are presented in Figure 10. In Figure 10a,b, significant GND accumulations are present along the grain boundaries and triple junctions, resulting from the abrupt slip paths at the grain boundaries [52,53]. However, the GND densities are lower within the grain interiors. The results are the same at the strain rate of 5 × 10^−3^ s^−1^ under RT, as shown in Figure 5a,b. The yellow rectangles in Figure 10b are the voids, the same as those shown in Figure 9b, marked with the red arrows. In Figure 10c,d, the twinning are presented as 5.85% within the part and 0.197% close fracture, similar to the strain rate of 5 × 10^−3^ s^−1^ under RT, as shown in Figure 5c,d. Therefore, at the low strain rate of 5 × 10^−5^ s^−1^, the tensile failure mechanics is mainly controlled by the dislocation movement along the slip path for MarBN steel under RT.

**Figure 11:**
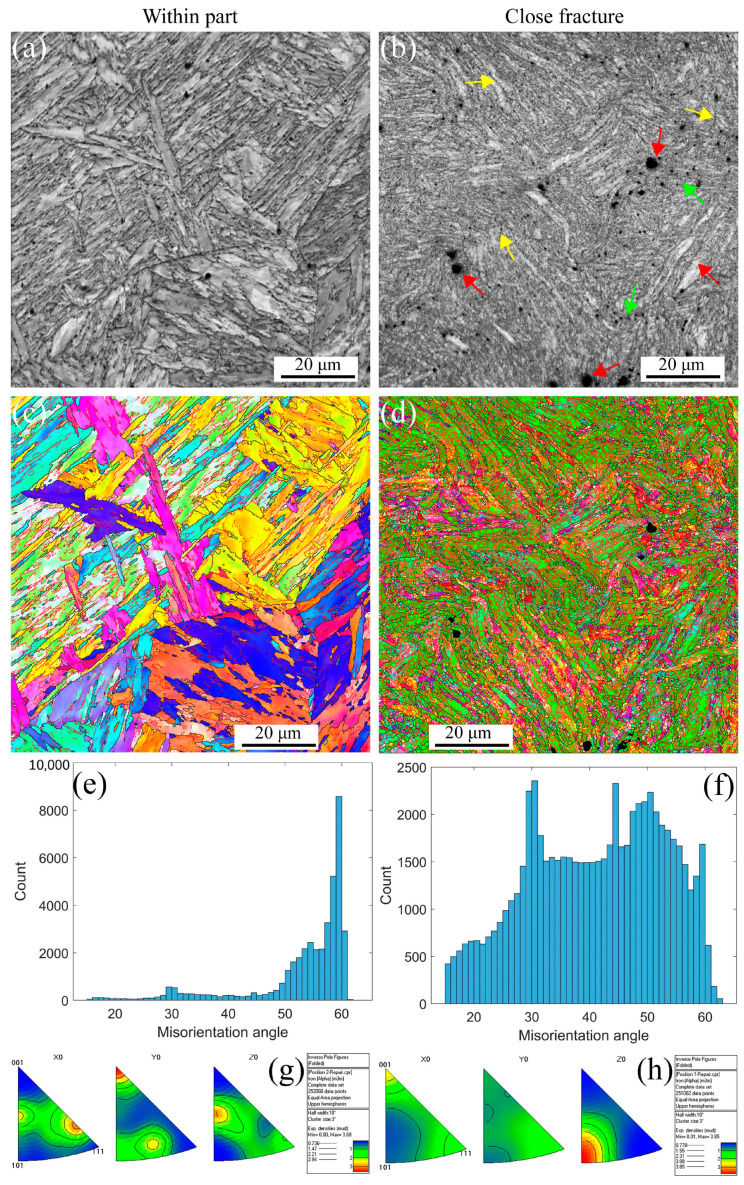
EBSD maps under different strain gradients at the strain rates of 5 × 10^−5^ s^−1^ under 430 °C (Red arrows indicate voids within the grain and along the line, green arrows indicate shrinking microstructures, and yellow arrows point to voids and shrinking cracks). (**a**,**b**) IQs; (**c**,**d**) IPFs; (**e**,**f**) misorientation angles; and (**g**,**h**) inverse polo figures.

Figure 11 shows the EBSD results at a strain rate of 5 × 10^−5^ s^−1^ under 430 °C. The stable and unstable PAGBs and martensite laths are observed within part and close fracture, respectively, as shown in Figure 11a–d. The internal defects, i.e., voids indicated with the red arrows, shrinkage microstructure defined with the green arrows, and cracks across the voids and shrinkage marked with the yellow arrows, are observed along with the distorted and elongated microstructure. The misorientation angles presented in Figure 11e,f are the same as the results under RT. However, it should be emphasized that this results from the combined influence of temperature and strain rate. In Figure 11g,h, the material orientation becomes uniform after high plastic strain during the uniaxial tensile process under 430 °C for MarBN. After high plastic strain, the texture is also present only in the <101>//Z_0_ direction, and the strength of the texture is enhanced compared to the low strain state.

**Figure 12:**
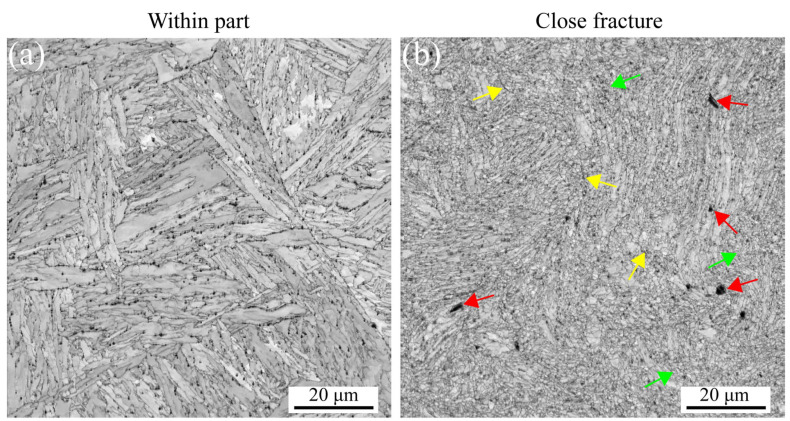

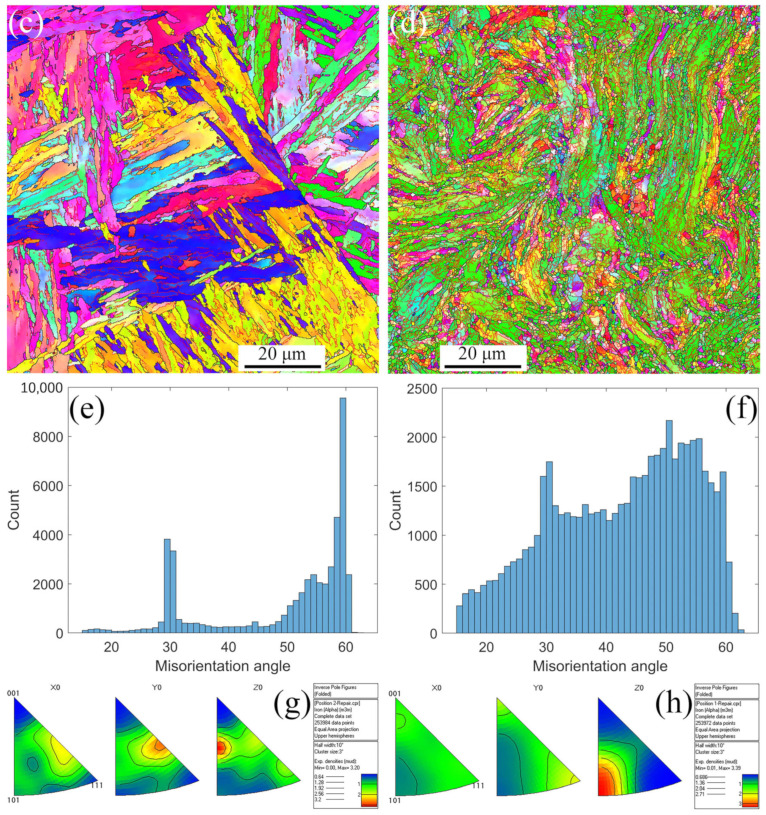
EBSD maps under different strain gradients at the strain rate of 5 × 10^−5^ s^−1^ under 630 °C (Red arrows indicate voids within the grain and along the line, green arrows indicate shrinking microstructures, and yellow arrows point to voids and shrinking cracks). (**a**,**b**) IQs; (**c**,**d**) IPFs; (**e**,**f**) misorientation angles; and (**g**,**h**) inverse polo figures.

**Figure 13:**
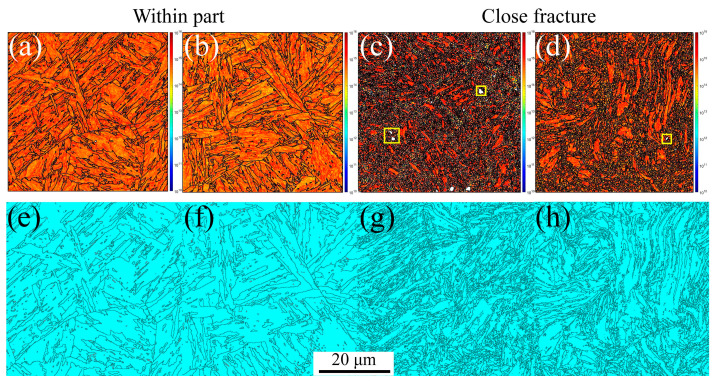
KAM, GND, and twinning maps under different strain gradients at the strain rates of 5 × 10^−5^ s^−1^ under 430 and 630 °C (Voids are inside the yellow square). (**a**–**d**) GNDs and (**e**–**h**) twinnings.

**Figure 14:**
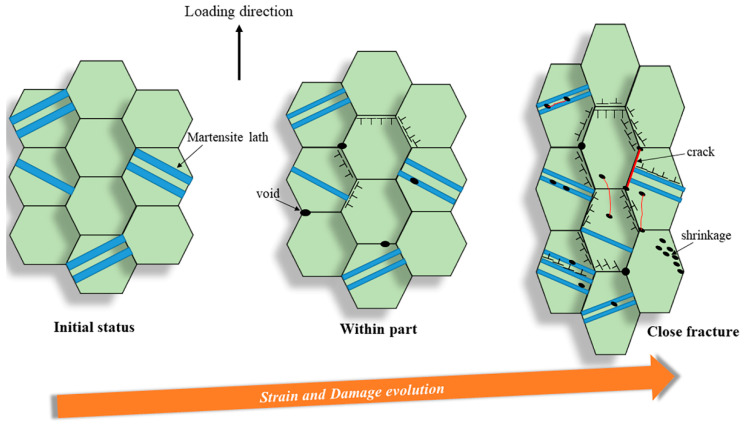
Schematic figure showing the failure mechanics at both strain rates under all temperatures.

Figure 12 shows the EBSD results at a strain rate of 5 × 10^−5^ s^−1^ under 630 °C. The results are the same as the results under RT and 430 °C. However, the apparent fine voids within the part appear along the grain boundaries due to the softening behavior under high temperatures [29,58,59]. On the other hand, the KAM, GND, and twinning maps are presented in Figure 13. The GND densities, as shown in Figure 13a–d, are the temperature and strain gradient balance. Moreover, the results are the same as the results under RT, indicating that the effect of the strain gradient on the GND densities is more critical than the temperature during tensile loading conditions. In Figure 13e–h, the twinning evolution is presented as 5.81% and 7.06% at 430 and 630 °C within the part, respectively, and 0.296% and 0.223% at 430 and 630 °C close fracture, respectively, indicating that with increasing strain, the twinnings decrease. The higher the temperature, the lower the number of twinnings, indicating that the number is related to the temperature and strain. Similar to room temperature and 430 ° C, only the <101>//Z_0_ direction texture exhibits significant texture with increasing strain, and the texture strength is significantly enhanced compared to low strain. To sum up, at a strain rate of 5 × 10^−5^ s^−1^ under all temperatures, from the macro-perspective, with an increasing strain, the microstructure becomes unstable and then constitutes the deformed and elongated grain boundaries, resulting in the formation of the three defects defined as void, shrinkage, and crack. From the micro-perspective, GND appears along the GBs and increases the dislocation density. These result in the formation of the LAGBs and decrease the number of twinnings. Under all strain rates and temperatures in this article, obvious textures are formed in the <101>//Z_0_ direction after significant plastic deformation. In addition, with the increase of plastic strain, the texture strength of almost all specimens increased significantly. This indicates that the changes in microstructure during the stretching process are dominated by plastic strain. However, at a strain rate of 5 × 10^−3^ s^−1^, the texture strength first weakens and then strengthens with the increase in temperature, which is strongest at 630 °C. However, when the strain rate is 5 × 10^−5^ s^−1^, the texture strength weakens with increasing temperature, and the texture is most obvious at RT. According to the stress–strain curves in Figure 1a,b, the plasticity of the material significantly improves with the decrease in strain rate under all three temperatures. This indicates that at the same temperature, the strain rate dominates plastic deformation, leading to different microstructural evolution. The strengthening sources of MarBN steel include grain boundary strengthening, dislocation interactions, and multiplication. Therefore, the failure mechanism for MarBN steel at both strain rates (5 × 10^−3^ s^−1^ and 5 × 10^−5^ s^−1^) under RT, 430, and 630 °C results from the interaction between the macro- and micro-behavior. The schematic figure is presented in Figure 14.

## 4. Constitutive Models

According to previous results, the plastic behavior of MarBN steel is associated with strain rate, temperature, and plastic strain evolution. Therefore, the constitutive models considering the strain rate, temperature, and work hardening should be addressed to describe and predict the true stress–true plastic strain accurately. In the present study, several constitutive models, including phenomenological (JC and KHL) and three physically based (PB, ZA, and VA) models, are compared systematically in flow stress during plastic deformation. The experimental results give the initial values of the model constants. After that, the model parameters can be fitted using the least square method. The $R^{2}$ values are used to evaluate the errors between the experimental and predictive results.

### 4.1. Phenomenological Models for JC and KHL

The plastic stress is given in the JC model [12] as follows,

(6) $$\sigma =\left(A+B\varepsilon^{n}\right)\left(1+C\ln{\dot{\varepsilon }}^{*}\right)\left(1-T^{*m}\right)$$

(7) $$T^{*}=\frac{T-T_{r}}{T_{m}-T_{r}}$$

where σ is the plastic stress, ε is the plastic strain; ${\dot{\varepsilon }}^{*}$ is the ratio of the strain rate and initial strain rate of 1 s^−1^; $T^{*}$ is the homologous temperature, which is determined by the applied temperature (T), reference ($T_{r}$), and melting temperatures ($T_{m}$); and A, B, C, *m* and n are the material constants. The fitting and error values of the JC model are given in Table 3. Under both strain rates and all temperatures, the descriptive errors of the JC model are very low. However, the predicted values at the strain rate of 5 × 10^−5^ s^−1^ are better than that at the high strain rate of 5 × 10^−3^ s^−1^.

Another phenomenological model of KHL [13,15,16] is used in the current work, in which the flow stress is expressed as follows,

(8) $$\sigma =\left[A+B{\left(1-\frac{\ln\dot{\varepsilon }}{\ln D_{0}}\right)}^{n_{1}}\varepsilon^{n_{0}}\right]{\left(\frac{\dot{\varepsilon }}{{\dot{\varepsilon }}^{*}}\right)}^{C}{\left(\frac{T_{m}-T}{T_{m}-T_{r}}\right)}^{m}$$

where ${\dot{\varepsilon }}^{*}$ is the reference strain rate; $D_{0}$ is the upper bound strain rate (10^6^ s^−1^); A, B, C, $n_{1}$, $n_{0}$, and m are the material constants; and the others are the same as in the JC model. The fitting and error values of the KHL model are presented in Table 4. For both the strain rates, the $R^{2}$ values at 630 °C are lower than those at RT and 430 °C. Thus, the KHL model is sensitive to temperature. Furthermore, the $R^{2}$ values at the high strain rate of 5 × 10^−3^ s^−1^ are higher than that at the low strain rate of 5 × 10^−5^ s^−1^, which indicates the KHL model can better predict the flow stress at the strain rate of 5 × 10^−3^ s^−1^. The model depends on the strain rate and temperature.

### 4.2. Physically Based Models of PB, ZA, and VA

The PB model [17,18,19,60], used to evaluate the flow stress in the current study, is defined as follows,

(9) $$\sigma =\sigma_{a}+\sigma^{*}=a\varepsilon^{n}+\hat{\sigma }{\left[1-{\left(-\frac{kT}{G_{0}}\ln\frac{\dot{\varepsilon }}{{\dot{\varepsilon }}_{0}}\right)}^{1/q}\right]}^{1/p}$$

where $\sigma_{a}$ and $\sigma^{*}$ are the athermal and thermally active parts of the flow stress; ${\dot{\varepsilon }}_{0}$ is reference strain rate, defined as 2.0 × 10^10^ s^−1^ [61]; p and q are material constants, defined as 0.5 and 1.5 [61], respectively; a, n, and $k/G_{0}$ are the material constants; $\hat{\sigma }$ is the threshold stress; $\dot{\varepsilon }$ is the strain rate; and ε is the plastic strain. The final fitting and error values are solved in Table 5. Compared with RT and 430 °C, the $R^{2}$ values at 630 °C are relatively low at both strain rates. In addition, the $R^{2}$ values at the high strain rate of 5 × 10^−3^ s^−1^ are higher than that at the low strain rate of 5 × 10^−5^ s^−1^, which means the PB model can describe the flow stress at the strain rate of 5 × 10^−3^ s^−1^. This model is dependent on the strain rate and temperature.

The second physically based model is the ZA model [21], in which the flow stress is expressed as follows,

(10) $$\sigma =C_{0}+C_{1}\exp\left(-C_{3}T+C_{4}T\ln\dot{\varepsilon }\right)+C_{5}\varepsilon^{n}$$

where $C_{0}$, $C_{1}$, $C_{3}$, $C_{4}$, and $C_{5}$ are the material constants; T is the applied temperature; and $\dot{\varepsilon }$ and ε are the strain rate and plastic strain, respectively. The final fitting and error values are presented in Table 6. At the same time, the $R^{2}$ values at the high strain rate of 5 × 10^−3^ s^−1^ are higher than that at the low strain rate of 5 × 10^−5^ s^−1^. In addition, the $R^{2}$ values under high temperatures are lower for both the strain rates than those under RT, especially at the strain rate of 5 × 10^−3^ s^−1^ under 430 and 630 °C. Therefore, the ZA model is more sensitive to the temperature and can better predict the flow stress at the strain rate of 5 × 10^−3^ s^−1^.

The third physically based model used in the present study is the VA model [20,62]. The flow stress is expressed as follows,

(11) $$\sigma =\hat{Y}{\left[1-{\left(\beta_{1}T-\beta_{2}T\ln\dot{\varepsilon }\right)}^{1/q}\right]}^{1/p}+B\varepsilon^{n}+Y_{a}$$

where $\hat{Y}$ is the threshold yield stress and $\beta_{1}$, $\beta_{2}$, B, $Y_{a}$, q, and p are the material parameters fitted with the initial values of the experimental values. The final fitting and error values are presented in Table 7. The values under 630 °C at a strain rate of 5 × 10^−5^ s^−1^ cannot be fitted using the VA model. However, the $R^{2}$ values at the high strain rate of 5 × 10^−3^ s^−1^ are higher than those at the low strain rate of 5 × 10^−5^ s^−1^. Therefore, the VA model is only valid for the strain rate of 5 × 10^−3^ s^−1^.

### 4.3. Verification and Comparison of Models

**Figure 15:**
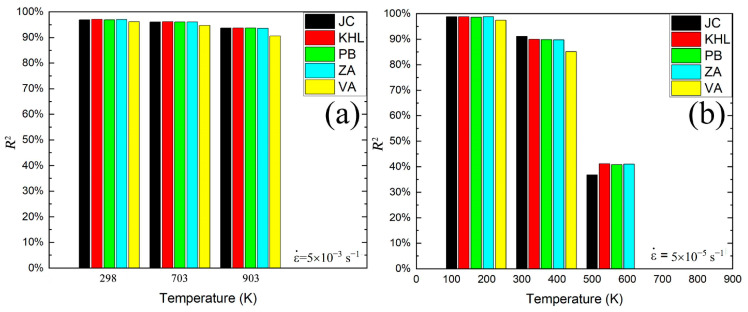
Comparison of different constitutive models under different temperatures at the high and low strain rates. (**a**) 5 × 10^−3^ s^−1^. (**b**) 5 × 10^−5^ s^−1^.

For comparison, the $R^{2}$ values for the different constitutive models between experimental and predicted results are plotted in Figure 15. At the high strain rate of 5 × 10^−3^ s^−1^, all the constitutive models present excellent error accuracy, as shown in Figure 15a. However, the error accuracy decreases with increasing temperature. It is noteworthy that only the VA model under all temperatures gives relatively low error accuracy. When the strain rate arrives at 5 × 10^−5^ s^−1^, the phenomenological models of JC and KHL present good accuracy under all temperatures compared to the other models. Under RT, all the models give good error accuracy. The error accuracy of the physically based models decrease with increasing temperatures, especially at 630 °C. Therefore, it is clear that all the constitutive models can predict the flow stress for both strain rates at RT. The predictive ability and error accuracy of these models (JC, KHL, PB, VA, ZA) at the low strain rate of 5 × 10^−5^ s^−1^ is better than that at the high strain rate of 5 × 10^−3^ s^−1^. However, for MarBN steel, the phenomenological models of JC (RT and 430 °C) and KHL (630 °C) give the best error accuracy in prediction under both strain rates due to the flexibility and minimum fitting parameters.

**Figure 16:**
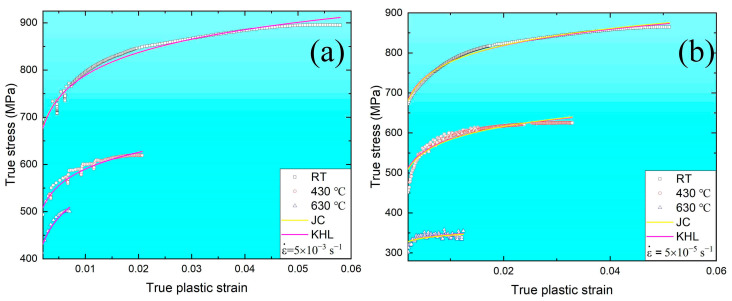
The predicted true stress–true plastic strain curves based on the JC and KHL models and experimental results at the strain rates of (**a**) 5 × 10^−3^ s^−1^ and (**b**) 5 × 10^−5^ s^−1^.

Furthermore, according to the previous results and analysis, Figure 16 shows the predicted true stress–true plastic strain curve based on the JC and KHL models at both strain rates under RT, 430, and 630 °C. Both models can descript the work-hardening behavior under RT and predict a softening effect of the strain rate and temperature on flow stress under 430 and 630 °C. However, the deviation in the simulated curves of these models from the experimental results is observed and associated with the serration behavior caused by the interaction between solute atoms and dislocations. The JC and KHL models only consider slip and twinning as the active deformation mechanisms during the plastic tensile process. Similar deviations have been reported for other alloys [63,64].

## 5. Conclusions

In the current paper, the thermomechanical behavior, microstructure, and constitutive models for MarBN steel are investigated at the strain rates of 5 × 10^−3^ s^−1^ and 5 × 10^−5^ s^−1^ under temperatures ranging from RT to 630 °C. Several conclusions can be obtained as follows:

1. Serrated flows are observed under all temperatures at the high strain rate of 5 × 10^−3^ s^−1^ (type C serrations) and under 430 and 630 °C at the low strain rate of 5 × 10^−5^ s^−1^ (type A+B serrations). This behavior is related to the strain rate and temperature for MarBN steel.
2. Work-hardenability is sensitive to the strain rate and temperature. At high strain rates of 5 × 10^−3^ s^−1^, the Holloman and Ludwigson equations provide a better prediction of tensile plastic properties. In contrast, at low strain rates of 5 × 10^−5^ s^−1^, coupling the Voce and Ludwigson equations appears to predict the flow relationship under RT, 430, and 630 °C.
3. At both strain rates, the microstructure results have the same evolutionary behavior based on the EBSD maps and analysis. From the micro-perspective, GND appears along the GBs and increases the dislocation density. These result in the formation of the LAGBs and decrease the number of twinning. The strengthening sources of MarBN steel include grain boundary strengthening, dislocation interactions, and multiplication. The failure mechanics are associated with void, shrinkage, and crack.
4. Several constitutive models (JC, KHL, PB, ZA, and VA) simulate the plastic flow stress under tensile experimental conditions. According to the experimental and simulation results, the predictive ability and error accuracy of these models (JC, KHL, PB, VA, ZA) at the low strain rate of 5 × 10^−5^ s^−1^ is better than that at the high strain rate of 5 × 10^−3^ s^−1^. For MarBN steel, the phenomenological models of JC (RT and 430 °C) and KHL (630 °C) give the best error accuracy in prediction under both strain rates due to the flexibility and minimum fitting parameters.

## Figures and Tables

**Table 1:** Detailed tensile properties under different temperatures at strain rates of 5 × 10^−3^ s^−1^ and 5 × 10^−5^ s^−1^.

Strain Rates /s^−1^	Temperature /°C	Yield Strength /MPa	Tensile Strength /MPa	Hardening Capacity
5 × 10^−3^	RT	694.85	895.81	0.29
	430	500.22	619.44	0.24
	630	414.17	501.43	0.21
5 × 10^−5^	RT	672.66	864.73	0.29
	430	450.93	625.20	0.39
	630	301.05	357.09	0.19

**Table 2:** Fitted values and error analysis for different flow relationships at the strain rates of 5 × 10^−3^ s^−1^ and 5 × 10^−5^ s^−1^.

Strain Rates /s^−1^	Temp /°C	*n*_1_	*n*_2_	*n*_3_	*n*_4_	*n*_5_	Holloman	Ludwik	Swift	Ludwigson	Voce
5 × 10^−3^	RT	0.0839	0.4977	0.09439	0.0839	72,946.0297	96.28%	89.86%	94.79%	96.28%	96.06%
	430	0.0874	0.5971	0.1089	0.2409	−22.3257	95.79%	91.34%	94.52%	96.70%	90.84%
	630	0.1297	0.9172	0.1925	0.2445	−61.8476	92.56%	83.4%	89.11%	93.50%	67.95%
5 × 10^−5^	RT	0.0752	0.4495	0.0870	0.0752	69,572.6375	98.16%	91.92%	96.26%	98.16%	98.70%
	430	0.0789	0.3177	0.0931	0.2339	−22.0530	89.80%	84.25%	85.85%	94.74%	96.45%
	630	0.0303	0.2453	0.0389	0.2421	−45.3192	36.45%	32.84%	31.98%	55.69%	12.64%

**Table 3:** Fitted values of the JC model under different temperatures at strain rates of 5 × 10^−3^ s^−1^ and 5 × 10^−5^ s^−1^.

Strain Rates/s^−1^	Temp/°C	*A*	*B*	*C*	*n*	*m*	*R* ^2^
5 × 10^−3^	RT	−1108.67	1581.09	−0.26	0.0201	79,943.93	96.94%
	430	−1452.34	1789.27	−0.28	0.0122	53.30	96.12%
	630	−2325.49	2563.60	−0.46	0.0073	312,616.49	93.68%
5 × 10^−5^	RT	−1105.53	1175.84	−1.42	0.0035	217.50	98.83%
	430	−900.05	1173.33	−0.20	0.0145	56.48	91.11%
	630	−503.54	775.43	−0.05	0.0097	34.53	36.79%

**Table 4:** Fitted values of the KHL model under different temperatures at strain rates of 5 × 10^−3^ s^−1^ and 5 × 10^−5^ s^−1^.

Strain Rates/s^−1^	Temp/°C	*A*	*B*	*C*	*n_1_*	*n_0_*	*m*	*R* ^2^
5 × 10^−3^	RT	−1152.67	748.90	−1240.53	1.39	0.0013	−550.50	97.12%
	430	−791.34	552.89	−12.82	1.56	0.0088	−5.56	96.14%
	630	−760.07	523.31	56.57	1.34	0.0044	−5.20	93.70%
5 × 10^−5^	RT	−1188.74	500.97	−33,793.68	1.69	0.0027	−413.46	98.82%
	430	−888.30	353.00	−479.67	1.73	7.44×10^−4^	−12.42	90.03%
	630	−446.72	233.92	30.43	1.76	0.0076	−1.46	41.18%

**Table 5:** Fitted values of the PB model under different temperatures at the strain rates of 5 × 10^−3^ s^−1^ and 5 × 10^−5^ s^−1^.

Strain Rates/s^−1^	Temp/°C	a	n	$\hat{\sigma }$	$k/G_{0}$	*R* ^2^
5 × 10^−3^	RT	3469.88	0.0219	−2988.45	3 × 10^−4^	96.93%
	430	3064.59	0.0181	−92332.69	6.09 × 10^−5^	96.10%
	630	2339.51	0.0315	−27848.31	2.57 × 10^−5^	93.70%
5 × 10^−5^	RT	3824.87	0.0164	−4207.28	2.43 × 10^−4^	98.70%
	430	5016.21	0.0094	−36506.49	6.56 × 10^−5^	89.85%
	630	422.32	0.0304	−1453.91	3.91 × 10^−5^	40.82%

**Table 6:** Fitted values of the ZA model under different temperatures at the strain rates of 5 × 10^−3^ s^−1^ and 5 × 10^−5^ s^−1^.

Strain Rates/s^−1^	Temp/°C	*C*_0_	*C*_1_	*C*_3_	*C*_4_	*C*_5_	*n*	*R* ^2^
5 × 10^−3^	RT	−563.03	−1444.74	8.76×10^−4^	−5.27 × 10^−4^	4241.46	0.0176	96.97%
	430	−65.09	−20,311.58	0.0056	2.98 × 10^−4^	2163.56	0.0267	96.06%
	630	−5086.28	−2.31 × 10^11^	−9.35 × 10^13^	1.45 × 10^18^	5912.99	0.0111	93.64%
5 × 10^−5^	RT	−16,031.26	−1.55 × 10^9^	−7.57 × 10^13^	1.78 × 10^17^	17,080.82	0.0035	98.81%
	430	−351.19	−427,663.99	0.005	2.50 × 10^−4^	3387.56	0.0142	89.75%
	630	107.55	−4778.18	0.0026	7.74 × 10^−6^	722.97	0.0166	41.04%

**Table 7:** Fitted values of the VA model under different temperatures at the strain rates of 5 × 10^−3^ s^−1^ and 5 × 10^−5^ s^−1^.

Strain Rates/s^−1^	Temp/°C	$\hat{Y}$	$\beta_{1}$	$\beta_{2}$	*p*	*q*	*B*	*n*	$Y_{a}$	*R* ^2^
5 × 10^−3^	RT	9.94	0.0019	2.84 × 10^−4^	0.2595	5.4996	1099.04	0.0909	65.12	96.19%
	430	6.95	9.74 × 10^−4^	8.22 × 10^−5^	0.3374	4.9660	673.83	0.2633	387.73	94.68%
	630	−1201.93	8.20 × 10^−4^	5.40 × 10^−5^	2.4681	0.6895	1137.98	0.3260	364.67	90.62%
5 × 10^−5^	RT	−19.35	0.0018	1.50 × 10^−4^	0.2680	5.0418	773.03	0.1278	347.37	97.52%
	430	25.82	8.63×10^−4^	5.62 × 10^−5^	0.3757	4.1880	534.08	0.2259	394.04	85.15%
	630									-

## Data Availability

The data that support the findings of this work are available from the corresponding author upon reasonable request.

## References

[1] Abe F., Tabuchi M., Semba H., Igarashi M., Yoshizawa M., Komai N., Fujita A. Feasibility of MARBN steel for application to thick section boiler components in USC power plant at 650 C. Proceedings of the 5th International Conference on Advances in Materials Technology for Fossil Power Plants.

[2] Abe F., Barnard P., Blum R., Chai G., de Barbadillo J.J., Di Gianfrancesco A., Forsberg U., Fukuda M., Hald J., Klöwer J. (2017). Materials for Ultra-Supercritical and Advanced Ultra-Supercritical Power Plants.

[3] Zhang X., Wang T., Gong X., Li Q., Liu Y., Wang Q., Zhang H., Wang Q. (2021). Low cycle fatigue properties, damage mechanism, life prediction and microstructure of MarBN steel: Influence of temperature. Int. J. Fatigue.

[4] Gong X., Wang T., Li Q., Liu Y., Zhang H., Zhang W., Wang Q., Wang Q. (2021). Cyclic responses and microstructure sensitivity of Cr-based turbine steel under different strain ratios in low cycle fatigue regime. Mater. Des..

[5] Wang Q., Wang Q., Gong X., Wang T., Zhang W., Li L., Liu Y., He C., Wang C., Zhang H. (2020). A comparative study of low cycle fatigue behavior and microstructure of Cr-based steel at room and high temperatures. Mater. Des..

[6] Zhang T., Wang X., Ji Y., Tang J., Jiang Y., Zhang X., Gong J. (2021). Cyclic deformation and damage mechanisms of 9%Cr steel under hybrid stress-strain controlled creep fatigue interaction loadings. Int. J. Fatigue.

[7] Fournier B., Sauzay M., Pineau A. (2011). Micromechanical model of the high temperature cyclic behavior of 9–12%Cr martensitic steels. Int. J. Plast..

[8] Sabzi M., Dezfuli S.M. (2018). Post weld heat treatment of hypereutectoid hadfield steel: Characterization and control of microstructure, phase equilibrium, mechanical properties and fracture mode of welding joint. J. Manuf. Process..

[9] Benaarbia A., Xu X., Sun W., Becker A., Jepson M.A.E. (2018). Investigation of short-term creep deformation mechanisms in MarBN steel at elevated temperatures. Mater. Sci. Eng. A.

[10] Zhang H., Wang Q., Gong X., Wang T., Pei Y., Zhang W., Liu Y., Wang C., Wang Q. (2021). Comparisons of low cycle fatigue response, damage mechanism, and life prediction of MarBN steel under stress and strain-controlled modes. Int. J. Fatigue.

[11] Xiao B., Xu L., Zhao L., Jing H., Han Y. (2017). Tensile mechanical properties, constitutive equations, and fracture mechanisms of a novel 9% chromium tempered martensitic steel at elevated temperatures. Mater. Sci. Eng. A.

[12] Johnson R., Cook W.K. A constitutive model and data for metals subjected to large strains high strain rates and high temperatures. Proceedings of the 7th International Symposium on Ballistics.

[13] Huang S., Khan A. (1992). Modeling the mechanical behaviour of 1100-0 aluminum at different strain rates by the bodner-partom model. Int. J. Plast..

[14] Abbasi-Bani A., Zarei-Hanzaki A., Pishbin M.H., Haghdadi N. (2014). A comparative study on the capability of Johnson–Cook and Arrhenius-type constitutive equations to describe the flow behavior of Mg–6Al–1Zn alloy. Mech. Mater..

[15] Khan A.S., Liang R. (1999). Behaviors of three BCC metal over a wide range of strain rates and temperatures: Experiments and modeling. Int. J. Plast..

[16] Khan A.S., Kazmi R., Farrokh B. (2007). Multiaxial and non-proportional loading responses, anisotropy and modeling of Ti–6Al–4V titanium alloy over wide ranges of strain rates and temperatures. Int. J. Plast..

[17] Nemat-Nasser S., Isaacs J. (1997). Direct measurement of isothermal flow stress of metals at elevated temperatures and high strain rates with application to Ta and TaW alloys. Acta Mater..

[18] Nemat-Nasser S., Guo W.-G. (2003). Thermomechanical response of DH-36 structural steel over a wide range of strain rates and temperatures. Mech. Mater..

[19] Nemat-Nasser S., Guo W. (2000). Flow stress of commercially pure niobium over a broad range of temperatures and strain rates. Mater. Sci. Eng. A.

[20] Voyiadjis G.Z., Abed F.H. (2005). Microstructural based models for bcc and fcc metals with temperature and strain rate dependency. Mech. Mater..

[21] Zerilli F.J., Armstrong R.W. (1987). Dislocation-mechanics-based constitutive relations for material dynamics calculations. J. Appl. Phys..

[22] (2016). Metallic Materials—Tensile Testing—Part 1: Method of Test at Room Temperature.

[23] (2018). Metallic Materials—Tensile Testing—Part 2: Method of Test at Elevated Temperature.

[24] Bachmann F., Hielscher R., Schaeben H. (2010). Texture Analysis with MTEX—Free and Open Source Software Toolbox. Solid State Phenom..

[25] Shen R.R., Efsing P. (2018). Overcoming the drawbacks of plastic strain estimation based on KAM. Ultramicroscopy.

[26] Arsenlis A., Parks D. (1999). Crystallographic aspects of geometrically-necessary and statistically-stored dislocation density. Acta Mater..

[27] Nye J.F. (1953). Some geometrical relations in dislocated crystals. Acta Metall..

[28] Rodriguez P. (1984). Serrated plastic flow. Bull. Mater. Sci..

[29] Choudhary B.K. (2013). Influence of strain rate and temperature on serrated flow in 9Cr–1Mo ferritic steel. Mater. Sci. Eng. A.

[30] Zhang L., Guo P., Wang G., Liu S. (2020). Serrated flow and failure behaviors of a Hadfield steel at various strain rates under extensometer-measured strain control tensile load. J. Mater. Res. Technol..

[31] Liu X., Fan J., Li K., Song Y., Liu D., Yuan R., Wang J., Tang B., Kou H., Li J. (2021). Serrated flow behavior and microstructure evolution of Inconel 625 superalloy during plane-strain compression with different strain rates. J. Alloys Compd..

[32] Chen C., Lv B., Feng X., Zhang F., Beladi H. (2018). Strain hardening and nanocrystallization behaviors in Hadfield steel subjected to surface severe plastic deformation. Mater. Sci. Eng. A.

[33] Lee S.-J., Kim J., Kane S.N., Cooman B.C.D. (2011). On the origin of dynamic strain aging in twinning-induced plasticity steels. Acta Mater..

[34] Karlsen W., Ivanchenko M., Ehrnstén U., Yagodzinskyy Y., Hänninen H. (2009). Microstructural manifestation of dynamic strain aging in AISI 316 stainless steel. J. Nucl. Mater..

[35] Qian L., Guo P., Zhang F., Meng J., Zhang M. (2013). Abnormal room temperature serrated flow and strain rate dependence of critical strain of a Fe–Mn–C twin-induced plasticity steel. Mater. Sci. Eng. A.

[36] Palaparti D.P.R., Choudhary B.K., Samuel E.I., Srinivasan V.S., Mathew M.D. (2012). Influence of strain rate and temperature on tensile stress–strain and work hardening behaviour of 9Cr–1Mo ferritic steel. Mater. Sci. Eng. A.

[37] Picu R.C., Vincze G., Gracio J.J., Barlat F. (2006). Effect of solute distribution on the strain rate sensitivity of solid solutions. Scr. Mater..

[38] Chaenock W. (1969). The initiation of serrated yielding at elevated temperatures. Philos. Mag..

[39] Hollomon J.H. (1945). Tensile Deformation. Met. Technol..

[40] Ludwik P. (1909). Elemente der Technologischen Mechanik.

[41] Swift H.W. (1952). Plastic instability under plane stress. J. Mech. Phys. Solids.

[42] Ludwigson D. (1971). Modified stress-strain relation for FCC metals and alloys. Metall. Trans..

[43] Voce E. (1948). The relationship between stress and strain for homogeneous deformations. J. Inst. Met..

[44] Voce E. (1955). A practical strain hardening function. Metallurgia.

[45] Reid F. (2000). The mathematician and the banknote: Carl Friedrich Gauss. Parabola.

[46] Tamhane A., Dunlop D. (2000). Statistics and Data Analysis: From Elementary to Intermediate.

[47] Sainath G., Choudhary B., Christopher J., Samuel E.I., Mathew M. (2015). Applicability of Voce equation for tensile flow and work hardening behaviour of P92 ferritic steel. Int. J. Press. Vessel. Pip..

[48] Choudhary B., Palaparti D.R., Samuel E.I. (2013). Analysis of tensile stress-strain and work-hardening behavior in 9Cr-1Mo ferritic steel. Metall. Mater. Trans. A.

[49] Choudhary B., Christopher J., Palaparti D.R., Samuel E.I., Mathew M. (2013). Influence of temperature and post weld heat treatment on tensile stress–strain and work hardening behaviour of modified 9Cr–1Mo steel. Mater. Des..

[50] Zhang H., Wang Q., Gong X., Wang T., Zhang W., Chen K., Wang C., Liu Y., Wang Q. (2021). Dependence on temperature of compression behavior and deformation mechanisms of nickel-based single crystal CMSX-4. J. Alloys Compd..

[51] Messerschmidt U. (2010). Dislocation Dynamics during Plastic Deformation.

[52] Jiang J., Britton T., Wilkinson A. (2013). Evolution of dislocation density distributions in copper during tensile deformation. Acta Mater..

[53] Ashby M. (1970). The deformation of plastically non-homogeneous materials. Philos. Mag. A J. Theor. Exp. Appl. Phys..

[54] Liu M., Wang Q., Cai Y., Lu D., Pei Y., Zhang H., Liu Y., Wang Q. (2022). Dependence on Manufacturing Directions of Tensile Behavior and Microstructure Evolution of Selective Laser Melting Manufactured Inconel 625. J. Mater. Eng. Perform..

[55] Wang L., Teng J., Liu P., Hirata A., Ma E., Zhang Z., Chen M., Han X. (2014). Grain rotation mediated by grain boundary dislocations in nanocrystalline platinum. Nat. Commun..

[56] Qin K., Yang L., Hu S.-S. (2014). Interpretation of strain rate effect of metals. Dynamic Behavior of Materials, Volume 1: Proceedings of the 2013 Annual Conference on Experimental and Applied Mechanics.

[57] Meyers M.A. (1994). Dynamic Behavior of Materials.

[58] Rae Y., Guo X., Benaarbia A., Neate N., Sun W. (2020). On the microstructural evolution in 12% Cr turbine steel during low cycle fatigue at elevated temperature. Mater. Sci. Eng. A.

[59] Eftink B.P., Vega D.A., El Atwani O., Sprouster D.J., Yoo Y.S.J., Steckley T.E., Aydogan E., Cady C.M., Al-Sheikhly M., Lienert T.J. (2021). Tensile properties and microstructure of additively manufactured Grade 91 steel for nuclear applications. J. Nucl. Mater..

[60] Rodríguez-Martínez J., Rusinek A., Klepaczko J. (2009). Constitutive relation for steels approximating quasi-static and intermediate strain rates at large deformations. Mech. Res. Commun..

[61] Xu Z., Huang F. (2013). Thermomechanical behavior and constitutive modeling of tungsten-based composite over wide temperature and strain rate ranges. Int. J. Plast..

[62] Voyiadjis G.Z., Almasri A.H. (2008). A physically based constitutive model for fcc metals with applications to dynamic hardness. Mech. Mater..

[63] Tiamiyu A., Tari V., Szpunar J., Odeshi A., Khan A. (2018). Effects of grain refinement on the quasi-static compressive behavior of AISI 321 austenitic stainless steel: EBSD, TEM, and XRD studies. Int. J. Plast..

[64] Wang H., Jeong Y., Clausen B., Liu Y., McCabe R., Barlat F., Tomé C. (2016). Effect of martensitic phase transformation on the behavior of 304 austenitic stainless steel under tension. Mater. Sci. Eng. A.

